# In reply to *Unopposed Estrogen and Endometrial Cancer: Association or Causation?*

**DOI:** 10.3325/cmj.2019.60.482

**Published:** 2019-10

**Authors:** Vladimir Trkulja, Pero Hrabač

In Reply: We thank LaCroix et al (1) for their comments on our text on odds ratio published in the February issue of the *CMJ* (2) and the concern that they expressed about the Croatian public health. They may rest assured that even first-year medical students in Croatia (not to mention health care professionals) are well aware of the causal relationship between estrogenic stimulation and endometrial carcinoma – just as we are, as it is obvious from our text on risk ratio (3), which followed in the April issue. We thank LaCroix et al for their interest in this series of texts on different types of studies and effect measures and their interpretation, and we hope they will continue to follow it.
